# How much does direct transmission between pigs contribute to Japanese Encephalitis virus circulation? A modelling approach in Cambodia

**DOI:** 10.1371/journal.pone.0201209

**Published:** 2018-08-16

**Authors:** Alpha Oumar II Diallo, Véronique Chevalier, Julien Cappelle, Veasna Duong, Didier Fontenille, Raphaël Duboz

**Affiliations:** 1 CIRAD, UMR ASTRE, Phnom Penh, Cambodia; 2 ASTRE, Univ Montpellier, CIRAD, INRA, Montpellier, France; 3 Epidemiology and Public Health Unit, Institut Pasteur du Cambodge, Phnom Penh, Cambodia; 4 UMR EpiA, INRA, VetAgro-Sup, Marcy l’étoile, France; 5 Virology Unit, Institut Pasteur du Cambodge (IPC), Phnom Penh, Cambodia; 6 Institut Pasteur du Cambodge (IPC), Phnom Penh, Cambodia; University of Minnesota, UNITED STATES

## Abstract

Japanese Encephalitis (JE) is the most important cause of human encephalitis throughout Asia and the Pacific. Although JE is a vector-borne disease, it has been demonstrated experimentally that transmission between pigs can occur through direct contact. Whether pig-to-pig transmission plays a role in the natural epidemiological cycle of JE remains unknown. To assess whether direct transmission between pigs may occur under field conditions, we built two mathematical models of JE transmission incorporating vector-borne transmission alone or a combination of vector-borne and direct transmission. We used Markov Chain Monte Carlo (MCMC) techniques to estimate the parameters of the models. We fitted the models to (i) two serological datasets collected longitudinally from two pig cohorts (C1 and C2) during two periods of four months on a farm on the outskirts of Phnom-Penh, Cambodia and to (ii) a cross-sectional (CS) serological survey dataset collected from 505 swine coming from eight different provinces of Cambodia. In both cases, the model incorporating both vector-borne and direct transmission better explained the data. We computed the value of the basic reproduction number *R*_0_ (2.93 for C1, 2.66 for C2 and 2.27 for CS), as well as the vector-borne reproduction number *R*_*pv*_ and the direct transmission reproduction number *R*_*pp*_. We then determined the contribution of direct transmission on *R*_*0*_ (11.90% for C1, 11.62% for C2 and 7.51% for CS). According to our results, the existence of pig-to-pig transmission is consistent with our swine serological data. Thus, direct transmission may contribute to the epidemiological cycle of JE in Cambodia. These results need to be confirmed in other eco-climatic settings, in particular in temperate areas where pig-to-pig transmission may facilitate the persistence of JE virus (JEV) during cold seasons when there are no or few mosquitoes.

## 1. Introduction

Japanese Encephalitis (JE) is endemic in Southeast Asia and the Pacific and is the most important cause of acute encephalitis in humans in these regions [1–5]; it is estimated that 3 billion people are at risk globally [3,6]. The annual incidence of JE is estimated to be at 68,000 cases in 24 countries with 13,600 to 20,400 deaths annually [2]. Japanese Encephalitis virus (JEV) is a *Flavivirus* of the family of *Flaviviridae*, which also includes Dengue, Yellow Fever, West Nile and Zika viruses [7,8]. JEV is a zoonosis, transmitted from pigs, the amplifying hosts, to human by mosquito bites. *Culex tritaeniorhynchus* is known to be the main vector [5,9,10]. It is established that Ardeid birds, such as Egrets and Herons are the wild reservoir host [11–14]. Viremia in humans and horses is insufficient to infect mosquitoes: they are accidental, dead-end hosts [3,5,10]. In a recent experimental study, it was shown that pigs were susceptible to oronasal infection and that vector free transmission between pigs can occur [15]. If pig-to-pig transmission occurs naturally on farms, it may contribute to the persistence of the virus, particularly when mosquito populations decline during the dry season in the tropics or during the winter in temperate areas. However, pig-to-pig transmission under field conditions, and its contribution to the epidemiological cycle of JE, is currently unknown.

Cambodia is a JE high-incidence country. A sentinel surveillance study on Japanese encephalitis in six Cambodian hospitals estimated the clinically-declared JE incidence in 2007 in the country at 11.1 cases per 100 000 children under 15 years of age [16]. JE is also highly endemic in pigs, with 95% of pigs older than 6 months of age seropositive for JEV when tested by IgG ELISA and hemagglutination inhibition tests in 2006 and 2007 [17].

For this study, we propose mathematical modelling to assess the importance of direct transmission between pigs in the epidemiology of JE in Cambodia. To our knowledge, few research papers on the mathematical modelling of the transmission dynamics of JEV have been published [18–21]. Most of these papers performed a mathematical analysis of JEV transmission by determining the basic reproduction number (*R*_0_, defined as the expected number of secondary cases produced by a single infection in an entirely susceptible population [22]), the possible equilibria (disease-free or endemic states) and discussed the stability properties of their model. Mukhopadhyay et al. used their model to simulate the seasonal fluctuations of JE transmission [23]. Another model was developed to investigate the effect of boosting immunity in humans against JE [24]. Naresh et al, proposed a nonlinear mathematical model accounting for demographic and environmental factors to analyze the effect of the environment on the transmission dynamics of JE considering varying human, reservoir and mosquito populations size [25]. Another compartmental model was built to describe JEV transmission dynamics in three districts in northwestern Bangladesh, where JE is endemic, in order to estimate the potential impact of pig vaccination on transmission [26]. The model suggested that vaccinating 50% of the total pig population each year resulted in an 82% reduction in the annual incidence of JE in pigs. None of these models has considered pig-to-pig direct transmission.

Here, we built two deterministic models of JE epidemiological cycle to assess the importance of pig-to-pig direct transmission of JE in Cambodia. The first model incorporates vector-borne transmission only. The second model assumes direct transmission between pigs combined with vector-borne transmission. We compare the fitness of the two models against three serological datasets collected from pigs in Cambodia to identify the model that best explains the empirical data. We then performed sensitivity analysis of the two models–vector borne transmission combined or not with direct transmission, to assess the influence of the model parameters on *R*_0_ and *I*_*pmax*_, the maximum number of infectious pigs. By decomposing *R*_0_ into pig-to-pig and vector-borne components, we quantified the relative contribution of direct transmission in the global transmission process under field conditions.

## 2. Method

### 2.1. Models

We built two deterministic models of the JE epidemiological cycle involving hosts (pigs) and vectors (*Culex* mosquitoes). The two models are:

- Vector Model (VM), which incorporates vector-borne transmission only,
- Vector and Direct transmission Model (VDM), which incorporates both pig-to-pig and vector-borne transmission.

We considered closed populations for pigs and mosquitoes.

The population of pigs is divided in four compartments, *M*_*p*_ for individuals with maternally derived antibodies, *S*_*p*_ for the susceptible individuals, *I*_*p*_ for the infectious individuals and *R*_*p*_ for the recovered individuals. In Cambodia, most of sexually matured sows are seropositive [17]. They give birth to piglets protected by maternal antibodies. When maternal antibodies disappear, piglets move from the compartment *M*_*p*_ to compartment *S*_*p*_. When infected by mosquito bites or by direct contact, susceptible pigs enter the *I*_*p*_ compartment. When the infectious period ends, infectious pigs enter the *R*_*p*_ compartment. Recovered pigs are considered to be immune to reinfection because of their short lifespan [27,28]. Pigs can produce high viremias 24 hours after infection, which can last up to four days [10]. Thus, we chose to omit the compartment “exposed” pigs because the incubation period define as the time from infection until signs and symptoms of the disease appear [29] is short compared with the duration of infectiveness and the average host life expectancy. Thus, infected pigs are treated as immediately infectious. The population of mosquitoes is divided in three compartments: *S*_*v*_,*E*_*v*_,*I*_*v*_ (Susceptible, Exposed and Infectious). We assumed that infectious mosquitoes do not recover [30]. Transmission from pigs to mosquitoes occurs when a susceptible mosquito bites an infectious pig. The susceptible mosquito (*S*_*v*_) then enters the exposed compartment *E*_*v*_ (infected but not yet infectious). After the latent period of 7–15 days, mosquitoes enter the compartment *I*_*v*_. They can transmit the virus when biting a susceptible pig.

**Fig 1:**
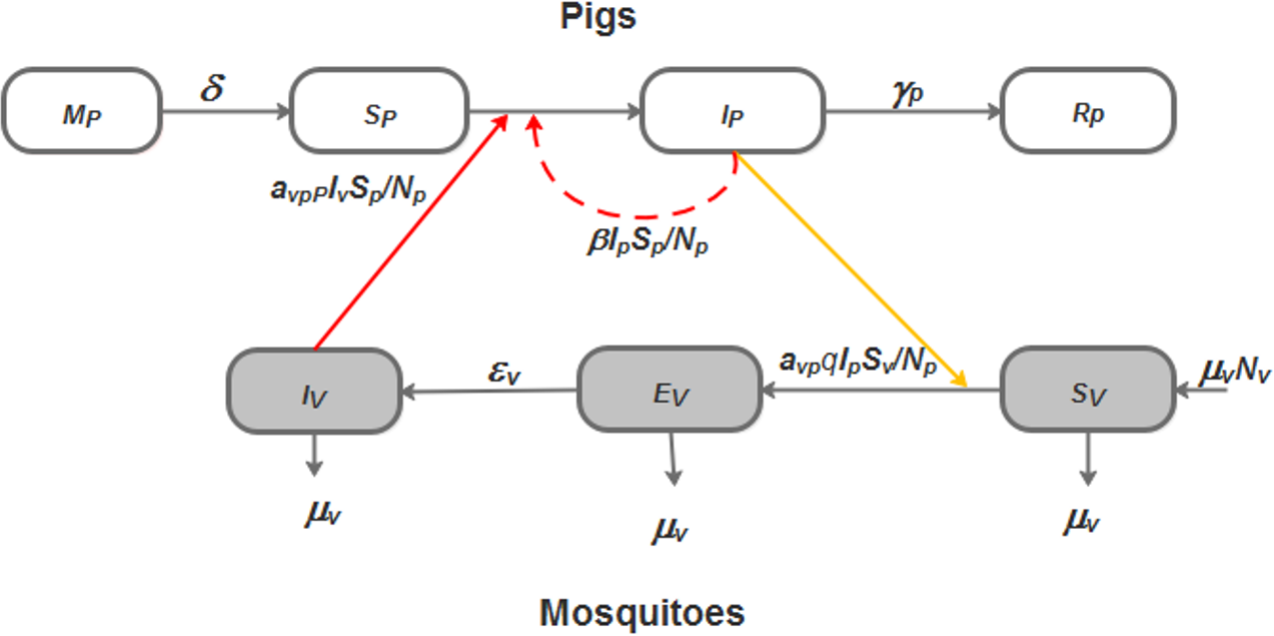
Flow diagram of the JE transmission models incorporating (VDM) or not (VM) pig- to -pig transmission. The white boxes represent the different health states of pigs. The grey boxes represent the different health states of vectors. Full dark arrows represent transition from one state to the other. The solid red arrow represents virus transmission between infectious mosquitoes and pigs. The solid orange arrow represents virus transmission between infectious pigs and susceptible mosquitoes. The red dashed arrow shows the direct transmission from pig-to-pig, for VDM only.

A viremic phase lasting 2–4 days has been described in pigs [15]. Given this short average infectious period for pigs compared to their lifespan, we did not consider births and deaths in the pig population. Conversely, since mosquitoes have a considerably shorter lifespan compared to pigs, and remain infectious for life, we introduced a demographic process, i.e. a renewal rate, for the vector population [30]. The assumed total number of pigs and mosquitoes were constant over time. JE vector population dynamic data are not yet available in Cambodia. Only one recent study performed in Kandal Province reports apparent peaks in abundance in May, July and December [31]. Therefore, we ignored the seasonal fluctuations of the mosquito population size.Fig 1 provides a graphic representation of both models (VM and VDM)

Parameters definitions and values are provided in Table 1.

**Table 1:** Parameter notation, definition and source of their values.

Parameters	Definition	Median value	Intervals	References
*δ*	Transfer rate from maternally protected to susceptible piglets, per day	0.5055	[0.011−1]	[31]
*β*	Transmission rate from infectious to susceptible pigs, per day	0.5	[0−1]	Assumed
*γ*_*p*_	Pig recovery rate, per day	0.375	[0.25−0.5]	[5,32]
*μ*_*v*_	Mosquito death rate, per day	0.04	[0.033−0.047]	[33,34]
*a*_*vp*_	Average daily biting rate per mosquito (average number of bites by mosquito and per pig), per day	0.25	[0.2−0.3]	[35]
*p*	Probability that an infectious mosquito transmits JEV to a susceptible pig when biting	0.205	[0.1−0.31]	[36]
*ε*_*v*_	Transfer rate from an exposed mosquito to an infectious mosquito, per day	0.103	[0.066−0.14]	[5,37]
*q*	Probability that a susceptible mosquito get infected when biting an infectious pig	0.56	[0.3−0.82]	[36]

### 2.2. Equations

The differential equations systems below formalize the models presented in Fig 1. The parameter values and their biological significance are provided in Table 1.

For the model considering vector-borne transmission only (VM), we have:

$$VM:\left\{ \begin{array}{l} \frac{{dM}_{p}}{dt}=\;-{\delta M}_{p}\\ \frac{{dS}_{p}}{dt}=\;{\delta M}_{p}-\frac{a_{vp}pI_{v}S_{p}}{N_{p}}\\ \frac{{dI}_{p}}{dt}=\;\frac{a_{vp}pI_{v}S_{p}}{N_{p}}-\gamma_{p}I_{p}\\ \frac{{dR}_{p}}{dt}=\;\gamma_{p}I_{p}\\ \frac{{dS}_{v}}{dt}=\;\mu_{v}N_{v}-\frac{a_{vp}qI_{p}S_{v}}{N_{p}}-\mu_{v}S_{v}\\ \frac{{dE}_{v}}{dt}=\;\frac{a_{vp}qI_{p}S_{v}}{N_{p}}-(\varepsilon_{v}+\mu_{v})E_{v}\\ \frac{{dI}_{v}}{dt}=\;\varepsilon_{v}E_{v}-\mu_{v}I_{v} \end{array}\right.$$ (1)

For the model combining pig-to-pig and vector-borne transmission (VDM), we have:

$$VDM:\left\{ \begin{array}{l} \frac{{dM}_{p}}{dt}=\;-\delta M_{p}\\ \frac{{dS}_{p}}{dt}=\;\delta M_{p}-\frac{a_{vp}pI_{v}S_{p}}{N_{p}}-\frac{\beta I_{p}S_{p}}{N_{p}}\\ \frac{{dI}_{p}}{dt}=\;\frac{a_{vp}pI_{v}S_{p}}{N_{p}}+\frac{\beta I_{p}S_{p}}{N_{p}}-\gamma_{p}I_{p}\\ \frac{{dR}_{p}}{dt}=\;\gamma_{p}I_{p}\\ \frac{{dS}_{v}}{dt}=\;\mu_{v}N_{v}-\frac{a_{vp}qI_{p}S_{v}}{N_{p}}-\mu_{v}S_{v}\\ \frac{{dE}_{v}}{dt}=\;\frac{a_{vp}qI_{p}S_{v}}{N_{p}}-(\varepsilon_{v}+\mu_{v})E_{v}\\ \frac{{dI}_{v}}{dt}=\;\varepsilon_{v}E_{v}-\mu_{v}I_{v} \end{array}\right.$$ (2)

With the following initial conditions:

$$M_{p}\left(0\right)=M_{0}\geq 0,\;S_{p}\left(0\right)=S_{0}\geq 0,\;I_{p}\left(0\right)=I_{0}\geq 0,\;R_{p}\left(0\right)=R_{0}\geq 0,\;S_{v}\left(0\right)=S_{0}\geq 0,\;E_{v}\left(0\right)=E_{0}\geq 0,\;I_{v}\left(0\right)=I_{0}\geq 0$$

The feasible set of systems (1, 2) is given by *D* = *D*_*p*_ × *D*_*v*_ (3) with:

$$D_{p}=\{\left(M_{p},S_{p},I_{p},R_{p}\right)|{M_{p}\geq 0,\;S_{p}\geq 0,\;I_{p}\geq 0,\;R_{p}\geq 0,}_{}\;M_{p}+S_{p}+I_{p}+R_{p}\leq N_{p}\}$$

$$D_{v}=\{{(S}_{v},E_{v},I_{v})|{S_{v}\geq 0,\;E_{v}\geq 0,\;I_{v}\geq 0,\;}_{}S_{v}+E_{v}+I_{v}\leq N_{v}\}.$$

The systems (1) and (2) are mathematically and epidemiologically well-defined because *D*_*p*_ and *D*_*v*_ are positively invariant (i.e. any trajectory of the system starting from an initial state in the positive orthant $R_{+}^{4}\times R_{+}^{3}$ remains in the positive orthant for all positive time) and each system has a unique solution that exist and remains in *D* for all positive time [38]. Note that the right-hand side of systems (1, 2) is Lipschitz continuous. Therefore, it exists a unique maximal solution for each system.

### 2.3. Datasets and model fitting

We fitted the VM and VDM to three different datasets. The first two datasets, contains serological results from longitudinal surveys performed on two cohorts (C1, S1 Table and C2, S2 Table) of fifteen pigs followed-up over two four-months periods from April to July 2014 and then from September 2014 to January 2015. These pigs were raised on a family farm located in the peri-urban environment of Phnom Penh, Cambodia. Blood samples were collected every 10 days from every pig from the age of two months, when maternal immunity is waning, to the age of six months, when pigs are usually sent to the slaughterhouse. In total, pigs in C1 and C2 were bled eleven and fourteen times respectively [31]. The third dataset contains the serological results of a cross-sectional (CS, S3 Table) survey performed in 505 pigs bred on family farms in Cambodia [17]. Three hundred and ninety three sera were collected in Kampong Cham Province in February 2006 and Kampong Speu province in July 2006. One hundred and twelve sera were collected in December 2007 in a slaughterhouse in Phnom Penh from swine that originated from eight different provinces in Cambodia. All pigs were aged from 1 to 24 months [17]. We arbitrarily distributed pigs among eleven age groups, from one to eleven months. The eleven month old group also included pigs over the age of eleven months since these pigs were all positive to JE.

We estimated the parameter values of VM and VDM using the Metropolis-Hastings method [39,40], and then selected the model that minimizes the sum of squares error when compared to the data [30,41,42]. The initial values of each parameter were the mean of their valid intervals given in Table 1. For C1 and C2, the number of piglets with maternal antibodies was obtained from Cappelle et al [31]. For CS Thus, the proportion of seropositive pigs was estimated for each class. For all the simulations, we assumed that, at time zero, pigs were either susceptible or had maternal antibodies. We initiated each simulation with the same proportion of infectious mosquitos. Table 2 provides the initial values for each model’s variables.

**Table 2:** Initial values of models’ variables for the fitting with the three dataset (C1, C2 and CS).

Dataset	*M*_*p*_	*S*_*p*_	*I*_*p*_	*R*_*p*_	*S*_*v*_	*E*_*v*_	*I*_*v*_
C1	13.0	2.0	0	0	999	0	1
C2	7.0	8.0	0	0	999	0	1
CS	0.36	0.64	0	0	0.999	0	0.001

As previously mentioned, data on JE mosquito vectors abundance and dynamics are lacking in Cambodia. Cappelle et al. performed mosquito trapping using a home-made Centers for Disease Control (CDC) light-trap. The trap was placed in the open-sided pig sty during the night preceding each blood sampling of the two cohorts. The researchers trapped 6,692 and 4,386 mosquitoes respectively during the 11 and 14 capture sessions performed between April and July 2014, and from September 2014 to January 2015 [31]. Therefore, for VM and VDM initial total number of mosquitoes (*N*_*v*_) was arbitrarily fixed to 1,000. Given the considerable uncertainty associated with this value, we assessed the effect of the variations of *N*_*v*_ by sensitivity analysis (SA) (see above).

In VM and VDM we noted identifiability issues between parameters *a*_*vp*_ and *p*; and between *a*_*vp*_ and *q*: *a*_*vp*_ multiplies *p* and *q*. It was, therefore, impossible to estimate *a*_*vp*_ and *q* or *a*_*vp*_ and *p* at the same time. Since *a*_*vp*_ has been previously estimated in [35], we fixed this parameter to its mean value (Table 1) to estimate *p* and *q*. We performed 50,000 iterations using the Monte Carlo Markov Chain algorithm.

### 2.4. Sensitivity analysis

Sensitivity Analysis (SA) ranks model’s parameters according to the effect of their value on model outputs. When a parameter is influential, the processes it summarizes are important regarding the dynamic of the model. We performed four SA for VM and VDM considering two outputs of interest: *R*_0_ and *I*_*pmax*_. We then compared the different SA results to analyze the impact of the introduction of direct transmission in the model on the ranking of parameters, and consequently the respective role of the processes they modelled.

At the initial stage of an epidemic, the transmission is directly related to *R*_0_. If *R*_0_ is greater than one, the disease can invade and spread through a naive population. The value of *R*_0_ is linked with the expected growth rate and the final total number of cases [43]. In a subdivided population with heterogeneous mixing, an explicit mathematical derivation of the final size of the epidemic as a function of *R*_0_ can be obtained, and it is possible to show that for the same value of *R*_0_, the final size of the epidemic is different from the homogeneous mixing hypothesis [44]. However, there is no explicit mathematical relationship between the final size of an epidemic and *R*_0_ for vector-borne diseases. An approximation of the upper bound can be determined in some cases [45], but the empirical relation between *R*_0_ and the final number of cases is still poorly understood. Consequently, we chose to consider *I*_*pmax*_ to characterize the dynamics of the model.

From Eq (1, 2), we derived *R*_0_ for VM and VDM using the Next Generation Matrix approach [46,47]. We noted *R*_*pp*_ the basic reproduction number for pig-to-pig transmission, and *R*_*pv*_ the basic reproduction number for vector-borne transmission. We have:

$$\mathrm{For}\;\mathrm{the}\;\mathrm{VDM}\;\mathrm{model}:\;R_{0}=\frac{R_{pp}+\sqrt{R_{pp}^{2}+4R_{pv}^{2}}}{2}$$ (3)

$$\mathrm{For}\;\mathrm{the}\;\mathrm{VM}\;\mathrm{model}:\;R_{0}=R_{pv},$$ (4)

$$\mathrm{with}:\;R_{pp}=\frac{\beta }{\gamma_{p}}$$ (5)

$$\mathrm{and}\;R_{pv}=\sqrt{\frac{a_{pv}^{2}pq\varepsilon_{v}N_{v}}{\gamma_{p}\mu_{v}(\mu_{v}+\varepsilon_{v})N_{p}}}$$ (6)

We performed an Analysis Of Variance (ANOVA) to compute the sensitivity indices of each model parameter, and the interactions between two parameters (i.e. simultaneous variation of their values) [48]. The indices reflect the proportion of the variance in *R*_0_ and *I*_*pmax*_ that is due to the variation of the parameter values. We then ranked them from the most to the least influential. We considered four levels for every parameter give in Table 1: their median values, their median values increased by 10%, and their median values increased and decreased by 20%. The vector-host ratio greatly influence the value of *R*_0_ [49,50]. To assess the influence of mosquito population abundance on transmission dynamics, we graphically analyzed the variation of *R*_0_ and *I*_*pmax*_. To focus on the other components of *R*_0_ and *I*_*pmax*_, we fixed the number of pig *N*_*p*_. We designed a complete factorial experimental plan resulting in 4^9^ possible combinations of parameters’ values. The sensitivity analysis was performed using the R statistical software (R version 3.2.3) with the package “aov” for the ANOVA [51].

### 2.5. Estimation of the contribution of pig-to-pig transmission in *R*_0_

We used the expression of *R*_0_ and the estimated parameters values of VDM to quantify *C*, the relative contribution of pig-to-pig transmission in VDM.

From Eq 3, we deduced that the necessary and sufficient condition to assert that *R*_0_ > 1 is $(R_{pp}+R_{pv}^{2})>1.$ Considering this inequality, we defined *C* as the proportion of the initial growth rate of pig-to-pig transmission in the total initial growth rate that is necessary to sustain an epidemic. It is defined as follow:

$$C=\frac{R_{pp}}{R_{pp}+R_{pv}^{2}}$$ (7)

## 3. Results

### 3.1. Model selection

**Fig 2:**
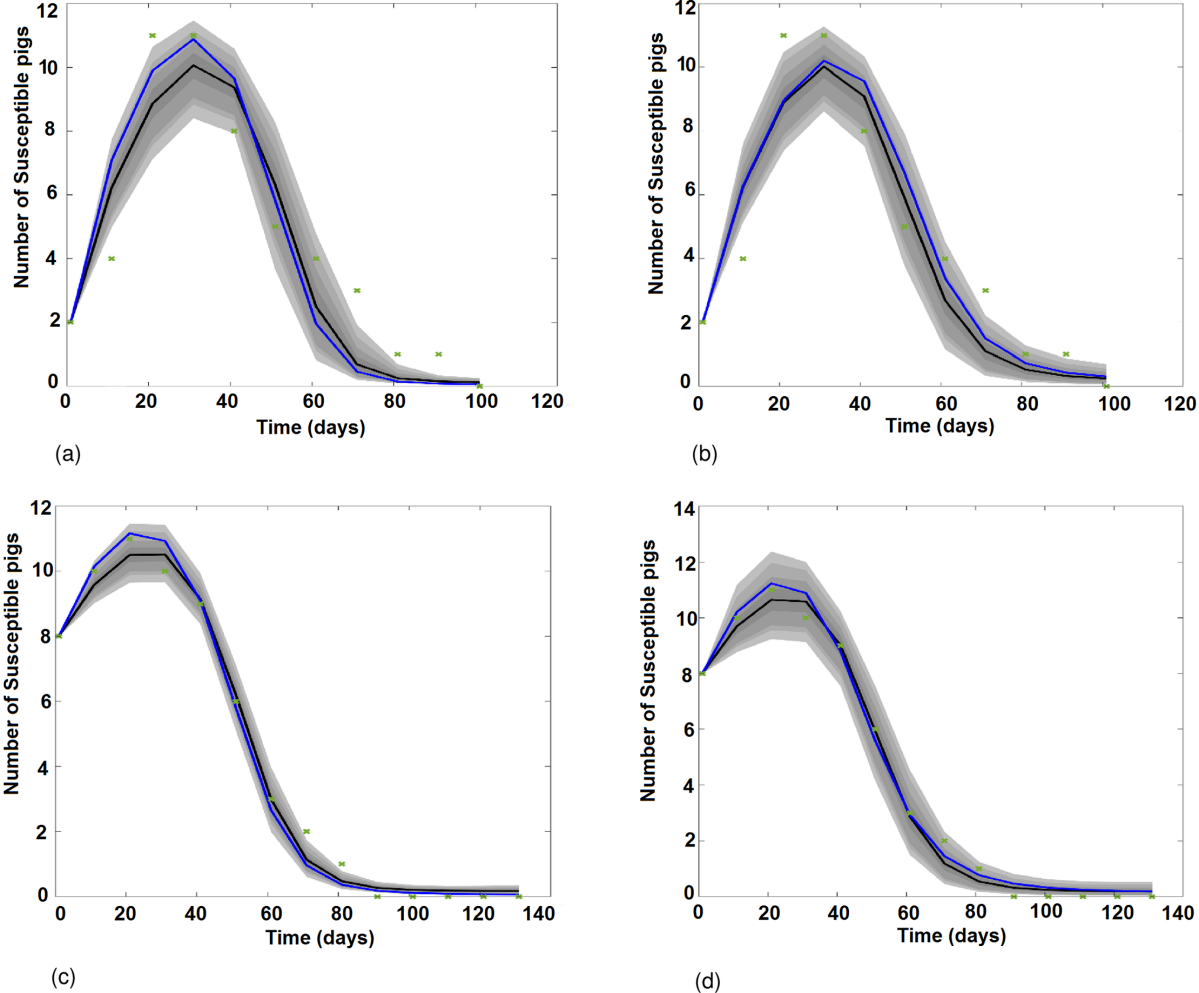
Results of the model fitting: Variation of the number of susceptible pigs with time as predicted by VM and VDM when fitting on cohort 1 (C1) and 2 (C2) data. (a) VM fitted to the C1 serological data. (b) VDM fitted to the C1 serological data. (c) VM fitted to the C2 serological data. (d) VDM fitted to the C2 serological data. Green crosses represent the number of susceptible pigs. The black solid line is the median fit; the blue solid line is the best fit. Grey areas with decreasing color intensity correspond to the 99%, 97%, 95% and 50% posterior limits of model uncertainty in predicting new observations.

Fig 2 presents the graphical results of the Vector Model (VM) and, the Vector and Direct transmission Model (VDM) fitting to the serological data obtained from pig cohorts 1 (C1, Fig 2A and 2B)) and 2 (C2, Fig 2C and 2D). Between day 0 (D0) and day 30 (D30), piglets are losing their maternal antibodies and become susceptible: the number of susceptible pigs increases. From D30 to D100, pigs are infected by JEV by mosquito bites in Fig 2A and 2C, and a combination of both vector borne and direct transmission in Fig 2B and 2D: the number of susceptible pigs decreases until 100% of them get infected.

**Fig 3:**
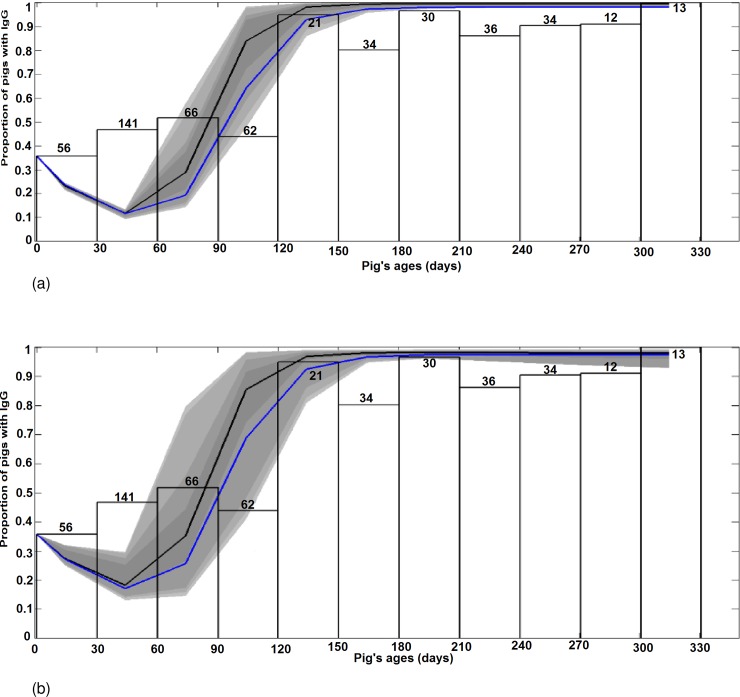
Results of model fitting: Variation of the proportion of seropositive pigs (maternally derived antibodies or natural infection) as predicted by VM and VDM when fitted to the CS serological data. (a) VM fits CS. (b) VDM fits CS. The y-axis is the proportion of pigs with IgG from maternal or infectious origins. The x-axis is the distribution of the proportion of seropositive pigs by age-class. The total number of individuals of each age-class is given on top of each corresponding white bar. The blue line represents the best fit. The black line shows the median fit. Grey areas with decreasing color intensity correspond to the 99%, 97%, 95% and 50% posterior limits of model uncertainty in predicting new observations.

Fig 3 presents the graphical results of VM and VDM fitted to serological data obtained from the cross sectional survey (CS). For pigs aged from 0 to 120 days, the shape of the curves slightly decreases, corresponding to the loss of maternal antibodies of piglets. Then, the proportion of seropositive pigs increases as pigs get infected, either through mosquito bites (Fig 3A), or a combination of vector borne and direct transmission (Fig 3B), and recover. From 120 days’ age and older, almost 100% of pigs are immune.

The Sum of Squares Error (SSE) and the estimated values of model parameters that best fit the three datasets are summarized in Table 3. For the three datasets, the VDM model has the smallest SSE. We note that parameters values are quite similar for all fittings, except for *β*.

**Table 3:** Sum of square error (SSE) and estimated parameter values of the models of best fit.

Dataset	Model	SSE	*δ*	*β*	*γ*_*p*_	*μ*_*v*_	*p*	*ε*_*v*_	*q*
C1	VM	26.40	0.05	-	0.48	0.03	0.15	0.08	0.47
	**VDM**	**18.42**	**0.04**	**0.41**	**0.5**	**0.04**	**0.11**	**0.07**	**0.48**
C2	VM	2.64	0.04	-	0.48	0.04	0.2	0.07	0.3
	**VDM**	**1.91**	**0.04**	**0.33**	**0.49**	**0.04**	**0.11**	**0.07**	**0.32**
CS	VM	0.34	0.03	-	0.45	0.05	0.12	0.09	0.31
	**VDM**	**0.27**	**0.02**	**0.17**	**0.48**	**0.04**	**0.10**	**0.08**	**0.30**

### 3.2. Sensitivity analysis

**Fig 4:**
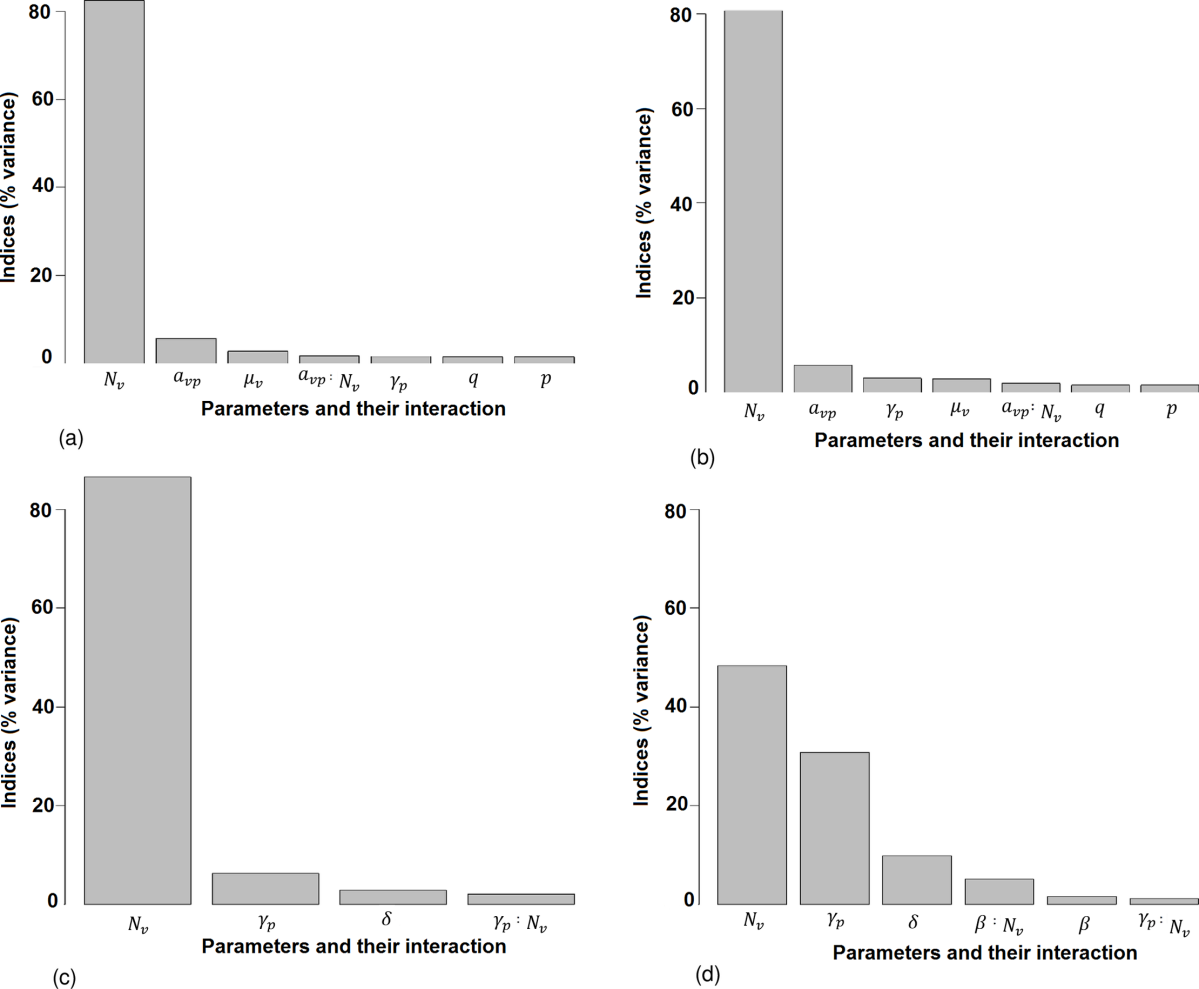
Results of the sensitivity analysis of *R*_0_ and *I*_*pmax*_ for models VM and VDM. (a) *R*_0_ for VM. (b) *R*_0_ for VDM. (c) *I*_*pmax*_ for VM. (d) *I*_*pmax*_ for VDM. The y-axis represents the percentage of the total variance explained by the variation of the parameters alone, or the interaction of two parameters. The parameters are ranked in decreasing order of the sensitivity indices, i.e. from the most to the least influential. We present only parameters responsible for more than 1% of the global variance.

Fig 4 presents the results of the sensitivity analysis for *R*_0_ and *I*_*pmax*_.

#### 3.2.1 Sensitivity analysis for *R*_0_

For both the VM and VDM, the number of mosquitoes (*N*_*v*_) is the most influential parameter, it generates more than 82% and 80% of the global variance of *R*_0_, respectively. The average daily biting rate per mosquito (*a*_*vp*_) is the second most influential parameter and participates in the global variance for 5.7% and 5.3% respectively. The mosquito death rate (*μ*_*v*_) participate 2.7% in VM and 2.8% in VDM. The pig recovery rate (*γ*_*p*_) contributes 1.62% of the variance for the VM compared to 2.92% for the VDM. The probability of transmission from an infectious pig to a susceptible mosquito (*q*) and the probability of transmission from an infectious mosquito to a susceptible pig (*p*) contributes 1.5% to the global variance for both models. The index of others parameters are less than 1%. Interactions between *a*_*vp*_ and *N*_*v*_ are responsible for 1.7% of the variance for the VM and 1.9% for the VDM. For both models, *N*_*v*_, *a*_*vp*_, *μ*_*v*_, *γ*_*p*_, *q*, *p* and their interactions represent more than 97% of the global variance.

#### 3.2.2 Sensitivity analysis for *I*_*pmax*_

As expected, when considering the VM *N*_*v*_ alone contributes more than 86% of the total variance of *I*_*pmax*_; whereas it contributes only in 48% for the VDM. *γ*_*p*_ contributes 6.4% for the VM compared to 30.8% for the VDM. For the VM *δ* (the transfer rate from piglets protected by maternally derived antibodies to susceptible pigs) contributes 2.9% to the total variance; whereas it represents 9.9% of the variance for the VDM. The transmission rate from infectious to susceptible pigs (*β*) contributes only 1.7% to the total variance in the VDM. The index values of others parameters are less than 1%. The interaction between *γ*_*p*_ and *N*_*v*_ is responsible for 2.1%. for the VM and 1.2 for the VDM. For the VDM the interactions between *β* and *N*_*v*_ contribute 5.1% of the total variance. Overall, for the VDM the *N*_*v*_, *γ*_*p*_, *δ*, and *β* parameters, and their interactions, represent more than 97% of the global variance.

### 3.3. Estimation of the contribution of pig-to-pig transmission in *R*_0_ for the model incorporating direct transmission (VDM)

The estimated values of *R*_0_ are very similar, i.e. 2.93, 2.66 and 2.27 when fitted to C1, C2 and CS respectively. The estimated values of *R*_*pv*_ are also greater than one, whereas the estimated values of *R*_*pp*_ are smaller than one. The estimated relative contributions of pig-to-pig transmission, *C*, are greater for C1 and C2 than for CS (Table 4)

**Table 4:** Estimated values of the basic reproduction numbers (*R*_0_, *R*_*pp*_, *R*_*pv*_) and *C*, the contribution of *R*_*pp*_ in *R*_0_ for the best model (VDM).

	C1	C2	CS
*R*_0_	2.93	2.66	2.27
*R*_*pv*_	2.48	2.29	2.02
*R*_*pp*_	0.83	0.69	0.35
*C*(%)	11.90	11.62	7.51

**Fig 5:**
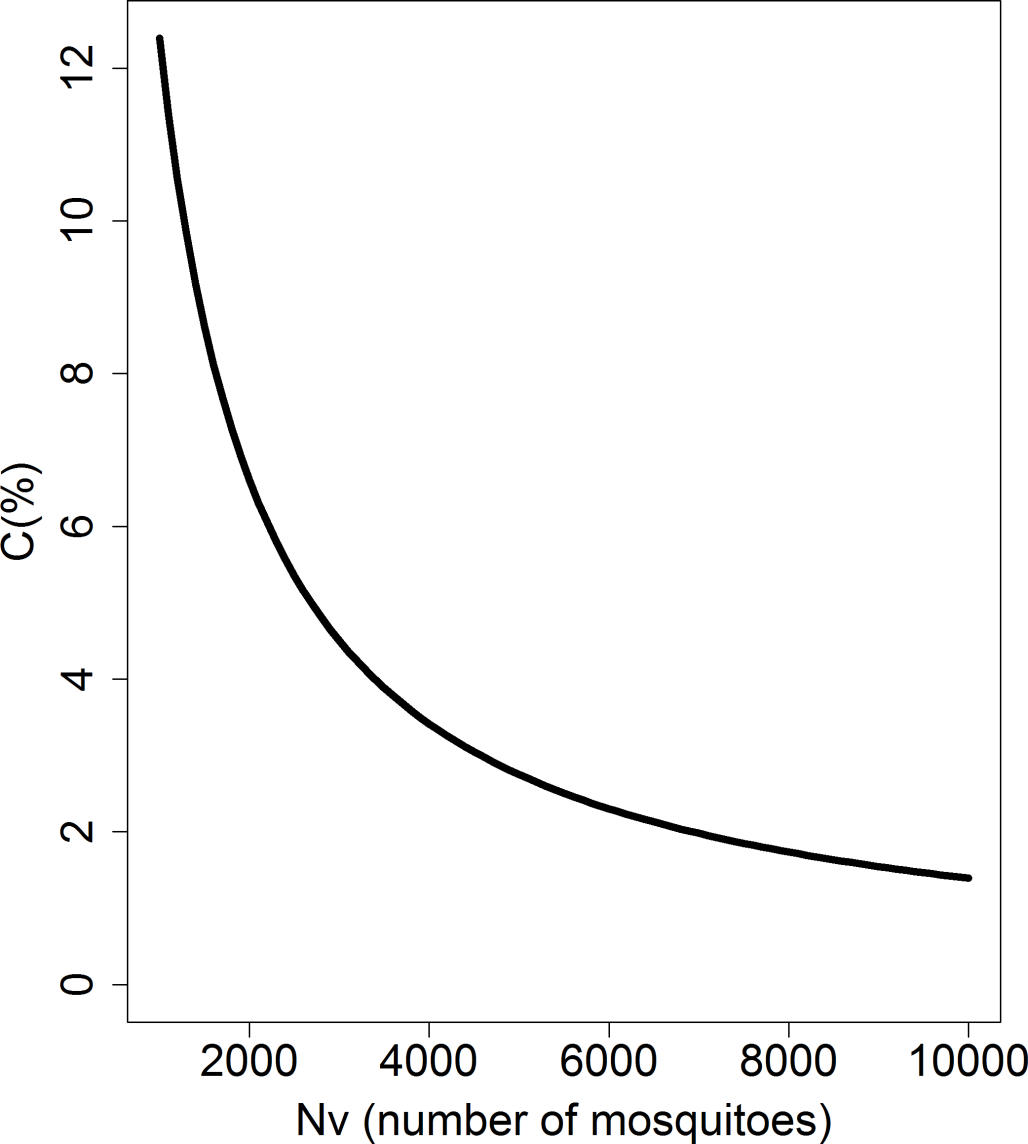
Influence of the number of mosquitoes (*N*_*v*_) on the value of direct transmission contribution (*C*). The x-axis represents the number of mosquitoes and the y-axis represents the contribution of direction transmission.

Plotting the impact of the number of mosquitoes on direct pig-to-pig transmission shows an inverse relationship, such that as *N*_*v*_ increases *C* decreases (Fig 5).

## 4. Discussion

Our model suggests that in the Cambodian context, the hypothesis of direct transmission of JEV between pigs cannot be rejected: the model including direct transmission between pigs (VDM) best described the empirical data of seroconversion of pigs to JEV on family farms across Cambodia. Prior to this study, direct transmission of JEV between pigs, under field conditions and where the disease is endemic, had not been tested. This is despite implications from a recent experimental study demonstrating that pigs may be infected with JEV through the oronasal route and that direct transmission of JEV between pigs can occur [15]. Our modelling approach provides a useful alternative to experimental studies to investigate the role of direct transmission of JEV between pigs under field conditions. The findings from this study may contribute to the understanding of the transmission dynamics and, potentially control, of JE, an important zoonosis, as discussed below.

As expected, sensitivity analyses for both models showed that the most influential parameter driving variations in *R*_0_ is the number of mosquitoes, *N*_*v*_. Consequently, there is an urgent need to improve our knowledge on JE vector dynamics and diversity in order to improve vector control strategies. However, when pig-to-pig transmission is introduced into the model the influence of *N*_*v*_ decreases by 37%, indicating that direct transmission from infectious to susceptible pigs may be substantial. Our models also show that the daily biting rate per mosquito, *a*_*vp*_ and the mosquito death rate, *μ*_*v*_ are important in the transmission process. Therefore, reducing both the ratio of mosquitoes per pigs and the contacts between mosquitoes and pigs, for example through mosquito nets as is the current practice on some family farms in Cambodia, would efficiently reduce JE transmission intensity within pig populations and consequently the risk for humans. Other than the first two most sensitive parameters (*N*_*v*_ and *a*_*vp*_), sensitivity indices diverge for VM and VDM. For VM, the next three most influential parameters are *μ*_*v*_, then the probability of transmission from an infectious pig to a susceptible mosquito (*q*) and the probability of transmission from an infectious mosquito to a susceptible pig (*p*). For VDM, the next three most influential parameters are *γ*_*p*_, the pig recovery rate, *q* and *p*. When pig-to-pig transmission is introduced in the model, the influence of *γ*_*p*_ increases: the slower the recovery of pigs the longer they remain infectious and the higher the chance they infect each other by direct transmission.

The results of the sensitivity analysis for *I*_*pmax*_ show that *N*_*v*_ is also the most influential parameter, for both VM and VDM. But for both models, *γ*_*p*_, the pig recovery rate becomes the second influential parameter. The third one is *δ*, the transfer rate from maternally protected to susceptible piglets : the higher *δ*, the fastest piglets can be naturally infected. Factors driving the length of *δ* are unknown. However, and in temperate countries where JE is seasonal, sow vaccination may increase the proportion of piglets born with maternal antibodies, thus help control seasonal JE circulation.

SA showed the remaining parameters had little influence on transmission dynamics, consequently with no implication for disease control.

Among the influential parameters, *N*_*v*_ and *a*_*vp*_ are the two parameters we could realistically act on in Cambodia to control *R*_0_ and *I*_*pmax*_ through vector control. Sow vaccination, would decrease the probabilities of transmission from an infectious pig to susceptible mosquito (*q*) and from an infectious mosquito to susceptible pig (*p*) [49,52,53]. However, because of the rapid turnover in pigs as a consequence of farming practices and the relative cost of vaccines, the implementation of a vaccination program would be challenging in the Cambodian context.

Considering the best model (VDM), estimations of *R*_0_, *R*_*pp*_, *R*_*pv*_, and *C* are of the same order of magnitude when fitted to C1, C2 and CS serological data. The mosquitoes/pigs ratio (*N*_*v*_/*N*_*p*_) has a strong influence on *R*_0_ and, therefore, on the transmission dynamics of the JEV in the pig population [49,50]. Increasing *N*_*p*_ with *N*_*v*_ held constant would decrease *R*_0_; increasing *N*_*v*_ with *N*_*p*_ held constant would increase *R*_0_. Consequently, the relative importance of pig-to-pig transmission on *R*_0_ is sensitive to *N*_*v*_ with *N*_*p*_ held constant: when *N*_*v*_ increases, the contribution of *R*_*pv*_ on *R*_0_ increases, with a reciprocal relationship. Therefore, a high density of mosquitoes combined with a high proportion of infectious mosquitoes could explain the fact that pig-to-pig transmission passes unnoticed. However, if the mosquito density is low, the basic reproduction number of vector-borne transmission *R*_*pv*_ and, therefore, *R*_0_ is low. In this circumstance, variations in the contribution of pig-to-pig transmission to variations in *R*_0_ would have a substantial effect on the total number of pigs acquiring infection. If *β* increases, then the basic reproduction number of pig-to-pig transmission *R*_*pp*_ and the contribution of *R*_*pp*_ to *R*_0_ also increases. Nonetheless, *R*_*pp*_ is below unity and *β* accounts for less than 1% in the variance of *R*_0_.

To assert that *R*_0_ > 1, the inequality $(R_{pp}+R_{pv}^{2})>1$ should be verified. In our model, the mechanisms represented by the parameters in *R*_*pv*_ are crucial to determining whether an epidemic may occur or not, as well as the slope of the initial growth phase. SA on *R*_0_ confirmed this assertion: the number of mosquitoes (*N*_*v*_) and the mosquito biting rate (*a*_*vp*_) are the most influential parameters for both VM and VDM. Furthermore, our results show that *R*_*pp*_ is always below unity, indicating that pig-to-pig transmission cannot alone sustain an outbreak.

There are several limitations to our exploratory work.

Firstly, the serological data from C1 and C2 were collected over limited periods of time, from April to July 2014 and from September 2014 to January 2015, in a small number of pigs and in the vicinity of Phnom Penh. Whereas, the CS serological data were obtained from a large cross-sectional survey, likely to represent the epidemiological pattern of JE in pigs for the whole country. Even if the epidemiological processes modelled are identical, i.e. vector-borne and direct transmission, the scale at which they occur (local vs national) and associated factors that may influence the mosquito vector abundance such as landscape or climate, are different. However, the parameter estimates from the models fitted to the three datasets are of the same order of magnitude for the best model, VDM. When considering the CS, estimates of *R*_0_, *R*_*pp*_, *R*_*pv*_, and *C* are smaller than for the models fitted to C1 and C2 data (Table 4). If it exists, direct transmission occurs only when there is close contact between pigs and is, therefore, less predominant when considering the whole transmission process at the regional scale.

Secondly, Cambodia has a tropical monsoon climate, with a rainy season from May to mid-November and a dry season from mid-November to April. This probably leads to a seasonal fluctuation in the population dynamics of mosquitoes. As noted above, Cappelle *et al* showed an “apparent peak of mosquito’s abundance in May, July and December” by trapping mosquitoes [31]; however, this trapping work was limited in time and space and does not represent the dynamic of JE vectors in Cambodia. Therefore, for our model we ignored the dynamics of the *Culex* population. Nevertheless, we graphically assessed the impact of increased mosquito abundance (*N*_*v*_) on the contribution of pig-to-pig transmission (*C*) (Fig 5). Increasing mosquito abundance reduces the component of pig-to-pig transmission (*C*) that is already weak in this study; but, according to *R*_*pp*_ formulation (Eq 5) and since *β* is not null, does not reduce it to zero. Thus, when considering that mosquito abundance fluctuations are a succession in time of maxima and minima, ignoring the mosquito population dynamics should not negatively affect the main findings of this study. This assumption will be assessed as soon as mosquito population dynamics become available.

Lastly, *Culex* mosquitoes are mainly ornithophilic: they feed on domestic birds such as chickens and ducks. An experimental study demonstrated that these birds produce a sufficient viremia to infect a mosquito when biting [54]. Furthermore, preliminary serological results obtained from chickens sampled in Vietnam and Cambodia (unpublished data) show that these domestic birds are exposed and produce antibodies against JE under natural conditions: domestic birds may be involved in JE epidemiological cycle. In our study, we have omitted these host species. Further studies are need to investigate the role of these potential secondary hosts in JE epidemiological cycle [55–57].

To our knowledge, this is the first study that investigates the existence of direct transmission between pigs under field conditions using mathematical modelling and serological data. The results obtained with the three sets of serological data used in this study showed that the hypothesis of the existence of a direct transmission of JEV between pigs cannot be ruled out. These results need to be confirmed (i) with a model incorporating mosquito abundance and population dynamic data and (ii) in other eco-climatic settings, in particular in temperate areas such as China and Japan, where transmission is seasonal [3,5]. In these areas, pig-to-pig transmission may facilitate JEV persistence during cold seasons. However, and as re-confirmed by our analysis, JE is primarily a vector-borne disease: in addition to human vaccination, vector control should be an alternative efficient way to reduce JE incidence in endemic countries [58,59]. Lastly, and whatever the eco-climatic condition, the dynamics of pig population renewal that determines the proportion of susceptible pigs is crucial to explaining the intensity of transmission of JE [10]. Adapted pig farm management strategies may interrupt virus circulation. In addition to human vaccination that efficiently protect humans but that not stop JEV circulation, alternative control methods could contribute in the forthcoming programs aiming at reducing the burden of JE in South East Asia.

## Supporting information

S1 Table Individual serological results from follow-up performed on fifteen pigs from April to July 2014 (C1) [31]. (XLSX)

S2 Table Individual serological results from follow-up performed on fifteen pigs from September 2014 to January 2015 (C2) [31]. (XLSX)

S3 Table Serological results of a cross-sectional survey performed in 505 pigs bred on family farms in Cambodia in 2006–2007 (CS) [17]. (XLS)
